# PDE Inhibitors and Autophagy Regulators Modulate CRE-Dependent Luciferase Activity in Neuronal Cells from the Mouse Suprachiasmatic Nucleus

**DOI:** 10.3390/molecules30153229

**Published:** 2025-08-01

**Authors:** Erik Maronde, Abdelhaq Rami

**Affiliations:** Faculty of Medicine, Institute for Anatomy II, Goethe University Frankfurt, Frankfurt am Main 60590, Germany

**Keywords:** phosphodiesterase, autophagy, 3-MA, PDE-inhibitors

## Abstract

Background: Signaling pathways like those depending on cAMP/PKA, calcium/calmodulin/CaMK, MEK-1/MAPK or PI3K/Akt have been described to modulate suprachiasmatic nucleus (SCN) neuronal signaling via influencing transcription factors like CREB. Here, we analyzed the effect of cyclic nucleotide phosphodiesterase inhibitors and structurally similar substances commonly used as autophagy modulators on a cell line stably expressing a cyclic nucleotide element-driven luciferase reporter. Methods: We used an SCN cell line stably transfected with a CRE-luciferase reporter (SCNCRE) to evaluate signaling and vitality responses to various isoform-selective PDE inhibitors and autophagy modulators to evaluate the mechanism of action of the latter. Results: In this study the different impacts of common PDE inhibitors and autophagy modulators on CRE-luciferase activity applied alone and in combination with known CRE-luciferase activating agents showed that (1) PDE3, 4 and 5 are present in SCNCRE cells, with (2) PDE3 being the most active and (3) the autophagy inhibitor 3-Methyladenin (3-MA) displaying PDE inhibitor-like behavior. Conclusions: Experiments provide evidence that, in addition to the extracellular signaling pathways components shown before to be involved in CRE-luciferase activity regulation like cAMP analogs, adenylate cyclase activators and beta-adrenoceptor agonists, cyclic nucleotide metabolism as realized by phosphodiesterase activity, or molecule/agents influencing processes like autophagy or inflammation, modulate transcriptional CRE-dependent activity in these cells. Specifically, we provide evidence that the autophagy inhibitor 3-MA, given that PDEs are expressed, may also act as a PDE inhibitor and inducer of CRE-mediated transcriptional activity.

## 1. Introduction

The second messenger adenosine-3′,5′-(cyclic)monophosphate (cAMP) is central for the elevation of CRE-dependent transcriptional activity in VIP-positive neurons in the nucleus suprachiasmaticus (SCN) of the mouse hypothalamus [1,2,3].

SCNCRE cells have been shown previously to display a robust response to cAMP-elevating agents like forskolin or β-adrenergic agonists [2,3]. However, it was also evident that temporal parameters like the onset and offset of activation or maximal response can vary between different agents especially if combinations of agents were tested.

These cells that are stably transfected with a cAMP-regulated element (CRE) luciferase reporter plasmid (named SCNCRE) are used here for pharmacological investigations of 1) phosphodiesterase inhibition as modulators of cAMP-levels and 2) agents/molecules potentially involved in the fine tuning of CRE-dependent transcriptional signaling in terms of additional or synergistic effects and the modulation of the time profiles like onset, maxima and offset characteristics.

Cyclic nucleotide phosphodiesterases (PDEs) are a family of enzymes catalyzing the split of 3′-5′phosphodiester bonds in signaling molecules like cAMP or cGMP to their corresponding 5′-phosphates as in 3′-5′cAMP to 5′-AMP. A role of PDEs in the physiology of the SCN has been proposed in studies in the hamster SCN [4,5].

To study the interaction of the cAMP system with other signal transductions systems, we specifically investigated isoform-specific phosphodiesterase inhibitors and molecules commonly used in cell biological studies on inflammation, apoptosis or autophagy (Tunicamycin, Rapamycin; 3-Methyl-Adenin/3-MA) [6,7,8,9,10,11,12].

As a base-modified nucleoside, 3-MA in particular suggests that it may influence phosphodiesterase activity and has already been shown, together with other methylated adenines, to inhibit PDE activity in rat pancreatic islet preparations [13], thereby contributing to cell survival [14].

In the context of cellular proliferation, differentiation and the related phenomena of apoptosis and autophagy, the relative impact of a substance like 3-MA on more than one signal transduction pathway (e.g., PI3K and PDE) could potentially lead to subtractive, additive or synergistic effects. Knowledge about such interactions may help unravel new ways to influence the affected processes and distinguish the different components involved. A certain overlap of structurally similar substances in terms of the similarity of their target structures has been noted before in the PDE-modulating action of cyclic nucleotide-dependent protein kinase modulators [15] or adenosine receptor agonists like caffein also inhibiting PDE activity [16].

In summary, we characterized the most common isoform-selective PDE inhibitors known to modulate hypothalamic neuronal cells and then explored molecules with nucleosidic or steroidal structural components for their ability to induce or modulate PDE-activity using a well-characterized hypothalamic suprachiasmatic CRE-reporter cell line [1,2,3].

It is interesting to note that the molecules we characterized here are commonly used agents in autophagy and inflammation research that so far have been rarely tested in the context of CRE-dependent transcriptional signaling [10,11,12].

## 2. Results

### 2.1. PDE Inhibitors

We first tested the potential influence of the PDE inhibitors Isobutyl-methyl-xanthine (IBMX; PDE1/general inhibitor), Milrinone (PDE3), Rolipram (PDE4) and Zaprinast (PDE5) on CRE-dependent transcriptional activity in SCNCRE cells. PDE inhibitors for the isoforms 3, 4 and 5 were chosen because of their expression in the brain/hypothalamus and their clinical relevance. IBMX was selected for its rather selective inhibition of PDE1, but also as a general PDE inhibitor targeting almost all known mammalian PDEs.

**Figure 1:**
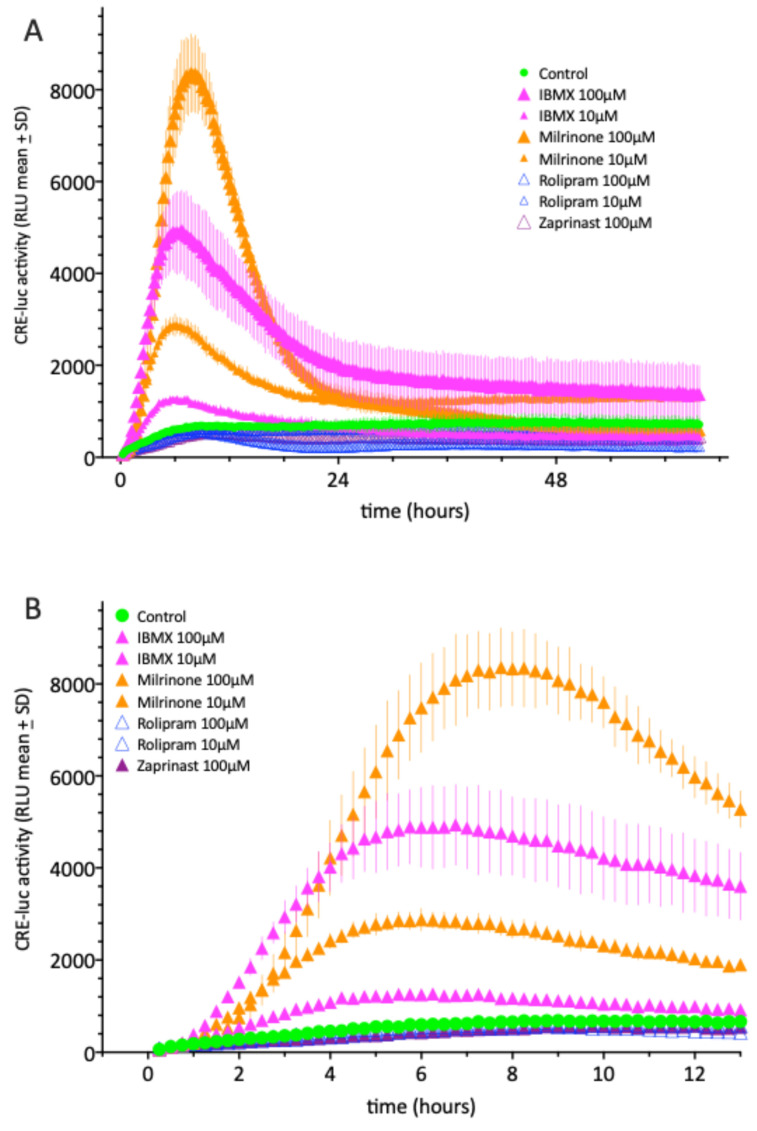
(**A**) CREluc activity after the application of 10 or 100 µM of the PDE inhibitors IBMX, Milrinone (PDE3), Rolipram (PDE4) and Zaprinast (PDE5) in comparison to a vehicle control over a time of 64 h. (**B**) presents the first 13 h of the data in (**A**) to show the different temporal profiles of IBMX and Milrinone. Shown are the means ± SD of N = 4 equally treated single wells in a 96-well multiwell plate. Data are representative of at least three similar experiments.

As seen in Figure 1, the application of 10 µM of IBMX or Milrinone is sufficient to elevate CREluc activity in SCNCRE cells, whereas Rolipram and Zaprinast applied alone do not exert elevation even at 100µM. The latter two substances even lead to RLU values below control levels at prolonged incubation times, indicating reduced cellular vitality. An additional finding is that IBMX application elevates CREluc activity more rapidly to a maximum, appearing approximately 2 h earlier than Milrinone (Figure 1B).

### 2.2. 3-MA and Other Autophagy Modulators

The effect of the autophagy inhibitor 3-methyl-adenine (3-MA) on SCNCRE cells is essentially unknown. Here, we tested concentrations in the most commonly used millimolar (mM; 10^−3^M) range dose-dependently.

**Figure 2:**
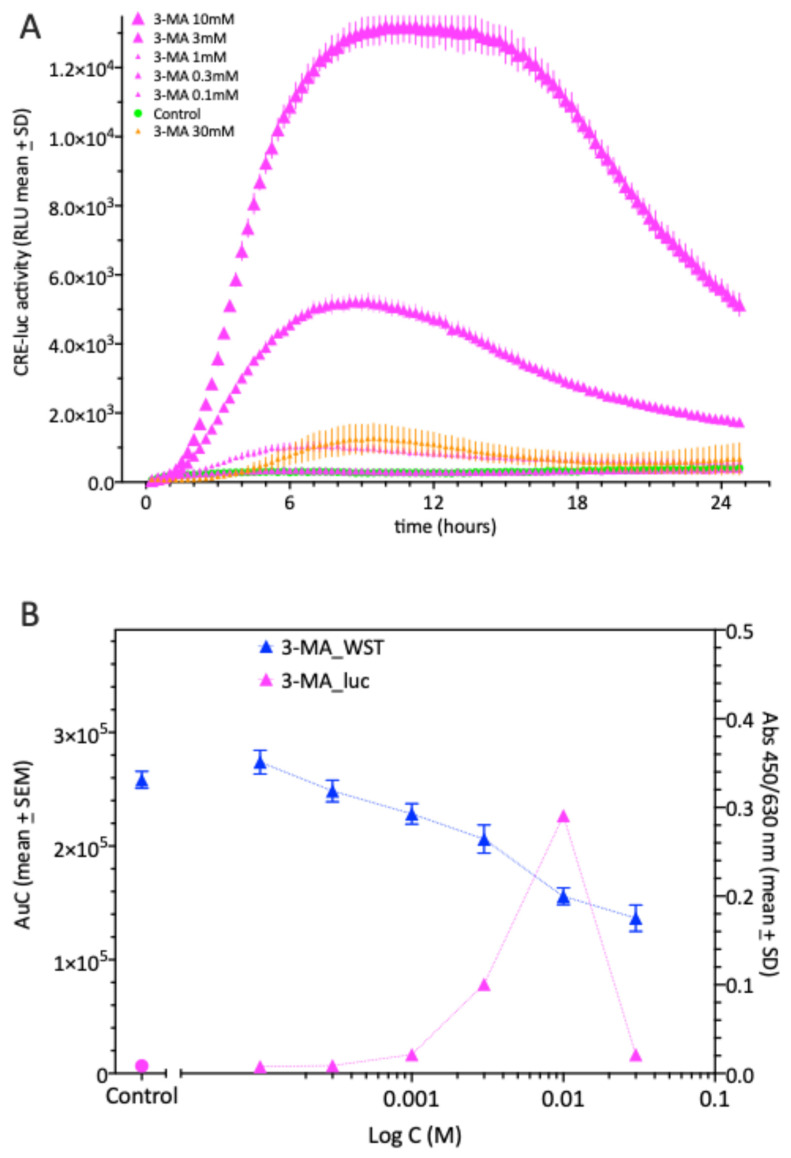
(**A**) CREluc activity after the application of different concentrations of 3-MA (magenta) in comparison to a vehicle control (green) over a time of 24 h. Shown are the means ± SD of N = 4 equally treated single wells in a 96-well multiwell plate. Note the striking difference between the doses of 10 and 30 mM of 3-MA. (**B**) Total CREluc- activity over 24 h (displayed as “area under the curve”: AuC; left y-axis) and WST-1 activity at 24 h (right y-axis) after the application of different doses of 3-MA (blue lines) to SCNCRE cells. The approximate EC_50_ for the luciferase activity is around 3 mM and appears as non-linear whereas the cell vitality decreases linearly with an IC_50_ also around 3 mM. Shown are the means ± SD of N = 4 equally treated single wells in a 96-well multiwell plate for WST-1 and the mean ± SEM of N = 4 for AuC.

Figure 2 shows that 3-MA increases CRE-luc activity (displayed at the left Y-axis in relative luminescence units: RLU) with a maximum at 10 mM and a steep decline at the highest tested concentration which was 30 mM.

On the right axis, we measured the vitality of the cells as judged by the WST-1 test, which depicts mitochondrial lactate dehydrogenase activity and becomes lower in absorption values with decreasing cellular vitality. Thus, in the test setup used above for the PDE inhibitors, 3-MA dose-dependently elevated CRE-luc activity, but shows signs of decreased cellular vitality with increasing 3-MA concentration at 24 h after starting the treatment.

Both of the autophagy activators, rapamycin and tunicamycin, do not cause a statistically significant change in the control (green) or isoproterenol (red) effect but display a tendency to increase the steepness of the onset of the isoproterenol curves. Shown are the means ± SD of N = 4 equally treated single wells in a 96-well multiwell plate.

**Figure 3:**
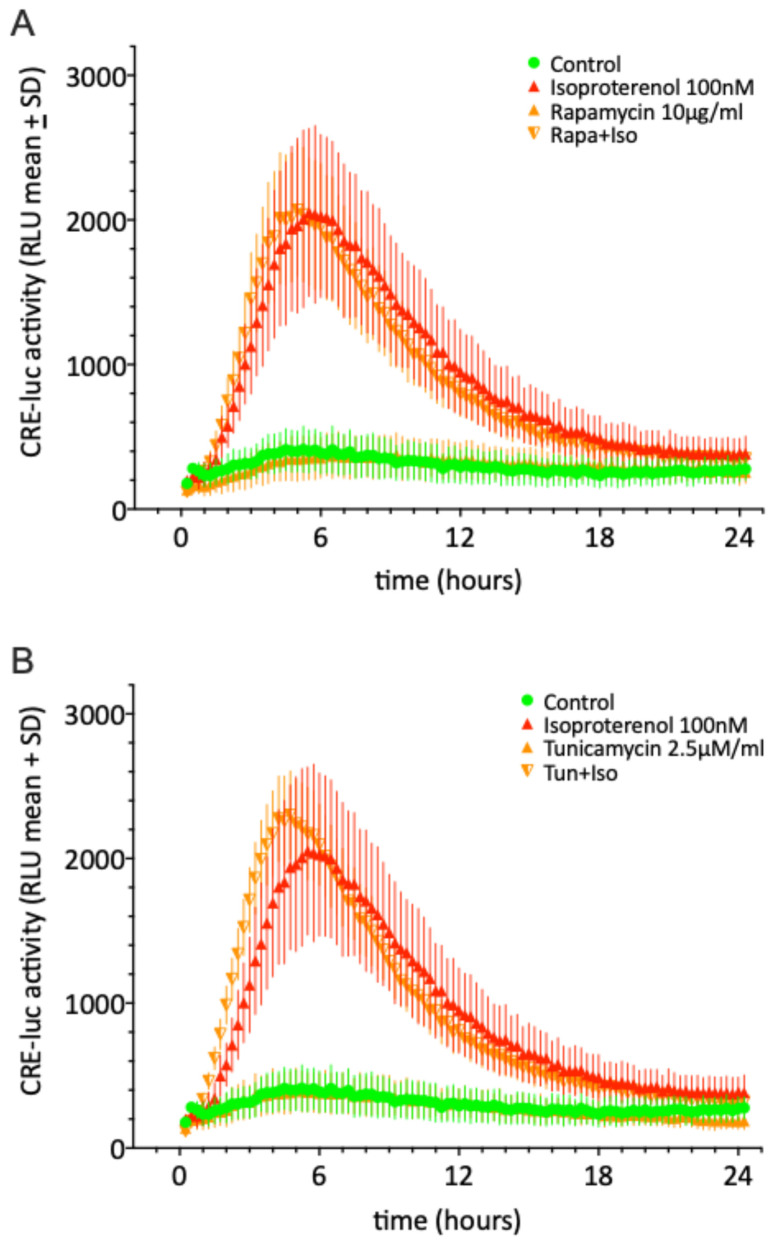
CREluc activity over 24 h and after the application of the autophagy activator rapamycin/sirolimus (**A**) or tunicamycin (**B**); orange half-triangles pointing down) with 100 nM of isoproterenol (α β1/2-adrenoceptor agonist; red line with triangle) to SCNCRE cells. Shown are the means ± SD of N = 4 equally treated single wells in a 96-well multiwell plate.

The autophagy activators rapamycin/sirolimus (upper graph) or tunicamycin (lower graph; orange half-triangles pointing down) do not cause a statistically significant change in the control or isoproterenol effect but display a tendency to increase the steepness of the onset of the isoproterenol curves (Figure 3).

**Figure 4:**
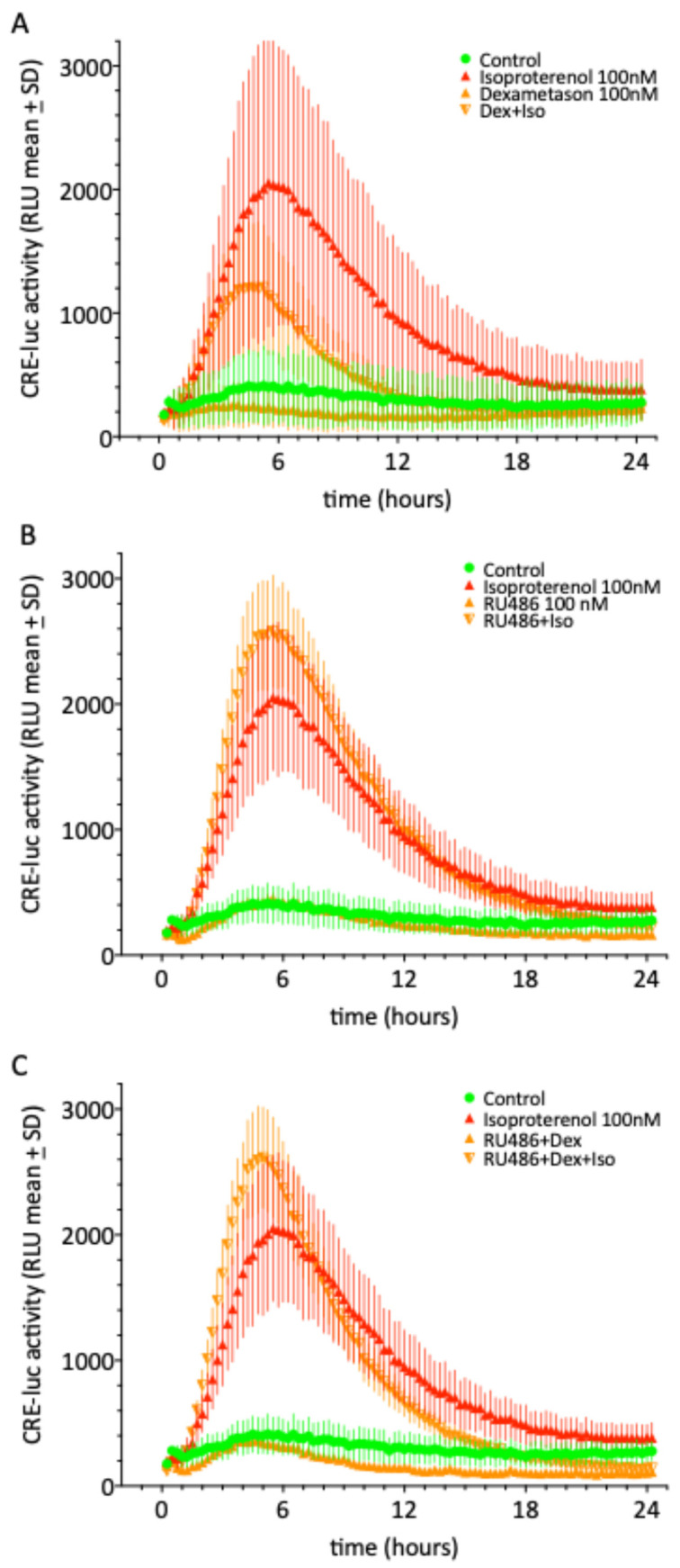
CREluc activity over 24 h after the application of the glucocorticoid receptor agonist and autophagy activator dexamethasone (**A**), the glucocorticoid receptor antagonist RU-486 (**B**) or the combination of both (**C**) with 100 nM of isoproterenol (α β1/2-adrenoceptor agonist; red line with triangle) to SCNCRE cells. Neither of the autophagy activators, dexamethasone nor its antagonist RU-486, nor their combined application cause a statistically significant change in the control (green) or isoproterenol (red) effect. However, the combination of dexamethasone and RU-486 displays a tendency to diminish below the control line, a sign of decreased cell vitality after prolonged application. Shown are the means ± SD of N = 4 equally treated single wells in a 96-well multiwell plate.

Neither the glucocorticoid receptor agonist and autophagy activator dexamethasone, nor the glucocorticoid receptor antagonist RU-486 or a combination of both cause a statistically significant change in the control or isoproterenol effect. However, the combination of dexamethasone and RU-486 displays a tendency to diminish below control levels, a sign of decreased cell vitality after prolonged application (Figure 4).

Rapamycin, tunicamycin, dexamethasone and RU-468 applied as single substances and in the concentrations used here did not cause a significant reduction in cellular vitality. However, the combination of dexamethasone and RU-486 did shown signs of reduced vitality after 6–8 h of treatment.

## 3. Discussion

### 3.1. General Discussion

The intracellular concentration of cAMP is determined by the basic synthesis rate of the adenylate cyclase/s, the availability of cytosolic ATP and the degradation of cAMP by 3′,5′phosphodesterases (PDE) [17,18,19,20]. It has been shown that PDEs can be modulated by many substances resembling nucleotide structures like caffein [16] or cyclic nucleotide modulators of PKA or PKG [15].

### 3.2. PDE Inhibitors

Cyclic nucleotide phosphodiesterases (PDEs) are a well-characterized family of enzymes expressed in different tissues in a wide variety of combinations and expression levels [21]. PDEs are also used as pharmaceutical agents with a wide clinical range influencing, e.g., cardial and pulmonary conditions [22,23]. The PDE3 inhibitor milrinone is in clinical use and also under development as a potentially memory-enhancing agent [24,25,26,27].

A frequently used inhibitor of PDEs which is used both as a broad range and a PDE1 inhibitor frequently is iso-butyl-methyl-xanthine (IBMX). We showed before that the application of IBMX leads to a fast and strong elevation of CRE-luciferase activity in SCNCRE cells which is additive to the action of forskolin [2]. Data also showed that the basal cAMP synthesis rate must be high in SCNCRE cells, because the application of moderate concentrations of IBMX alone (10 to 100 µM) rapidly and strongly increased CREluciferase-dependent luminescence.

Based on this previous finding, we sought to investigate the PDEs of SCNCRE cells in more detail. We confirmed the ability of IBMX to elevate CRE-luc activity in SCNCRE cells and extended this knowledge with the finding that the PDE3 inhibitor milrinone is about equally as effective as IBMX is. The PDE4 inhibitor rolipram [28] and the PDE5 Zaprinast [29] did not show CRE-luc elevation applied alone up to 100µM, but added to the effect of isoproterenol, a ß-adrenoceptor agonist as expected for PDEs present but with rather low expression and activity levels. The high activity of PDE3 (together with that of PDE1) might also have clinical implications. Since the SCNCRE cells used here are derived from the suprachiasmatic nucleus of the mouse and a high expression and activity level could translate into human patients, that application of milrinone due to any one of the above-mentioned conditions may also influence their biological clock and processes regulated by that, like, for example, sleep.

### 3.3. Autophagy Modulators

Autophagy is the process of cellular degradation and the recycling of a cell’s intrinsic structures [30]. For the inhibition of autophagy, among many others, 3-methyl-adenin (3-MA) is widely used [9]. 3-MA is a well-described Class III PI3Kinase inhibitor and it is widely assumed that this is the mechanistic reason for its inhibition of autophagy as well.

The effect of 3-MA on SCNCRE cells is essentially unknown. However, in the test setup used here for the PDE inhibitors, 3-MA dose-dependently elevated CRE-luc activity. Thus, an autophagy inhibitor like 3-MA strongly resembling the core structure of many other PDE modulators is suggestive of a function as a PDE modulator/inhibitor and is shown here to include CRE-luc activity in a mouse hypothalamic cell line. Another inhibitor of PI3K is LY-294002 which reportedly inhibits autophagic sequestration [31]. Interestingly, LY294002 application in our setup also further elevated isoproterenol-induced SCNCRE-luc activity (Supplementary Figure S2).

Interestingly, a similar observation that 3-MA (and similar molecules) displays PDE-inhibitor activity has been made before in rat islet cell preparations [13]. Among the observations in this work was that 3-MA dose-dependently inhibited PDE activity in the concentration range (millimolar) in which many studies before have used 3-MA as a selective, non-ATP-depleting agent to inhibit autophagy [9,10,11,12].

As of June, 2025, there are 4084 PubMed entries with the search terms “3-Methyladenine/3-MA and autophagy” which renders the PDE-inhibitory property of 3-MA a relevant issue.

In SCNCRE cells, 3-MA dose-dependently elevated CRE-luc activity with an EC_50_ at approximately 1–3 mM. At this concentration, the cell vitality (after the end of the experiment = 24 h after the start of treatment) is already significantly reduced. However, it has to be taken into account that the maximum CRE-luc activity for 3-MA was reached at 8 h and the processes reducing the vitality may not have started or were not yet influencing reporter gene activity.

Autophagy activators (Rapamycin, tunicamycin and dexamethasone) as well as the glucocorticoid receptor antagonist RU-468 applied as single substances and in the concentrations used here did not significantly influence CRE-luc activity in SCNCRE cells. Rapamycin apparently exerts its autophagy-activating effect by inhibiting the serin kinase named “mammalian target of rapamycin” or mTOR, which terminates autophagy and supports the reformation of lysosomes [32]. Tunicamycin induces endoplasmatic (ER) stress, thereby triggering autophagy [33]. Tunicamycin is not primarily classified as a direct autophagy activator, but it can indirectly induce autophagy as part of the cellular stress response, particularly endoplasmic reticulum (ER) stress. Tunicamycin inhibits N-linked glycosylation in the ER, leading to the accumulation of misfolded proteins. This triggers the unfolded protein response (UPR), which can activate autophagy as a downstream effect to alleviate stress [34]

Dexamethasone is an anti-inflammatory glucocorticoid which induces autophagy in most analyzed cell lines and has recently been shown to contribute to the paracrine enhancement of BDNF maturation, thereby supporting neuronal survival [35]. RU-486 or mifepristone is mostly known as an antagonist for the progesterone receptor but can also efficiently act as an antagonist for glucocorticoid action [36]. Here, neither dexamethasone nor RU-486 nor the combination of both significantly influenced CRE-luc activity.

Rapamycin, tunicamycin, dexamethasone and RU-486 also did not cause a significant reduction in cellular vitality, although the combination of dexamethasone and RU-486 did shown signs of reduced vitality after 6–8 h of treatment. There are visible but not statistically significant effects on the onsets, maxima and offsets of the isoproterenol temporal profiles that may merit more detailed investigation, but these will not be further elaborated here.

Finally, an important more recent aspect of autophagy is “autophagic flux” [37,38], which is the emphasis on autophagy as also a temporal phenomenon. The method used here follows the temporal development of the CRE-luc activity in SCNCRE cells with a 15 min temporal resolution over 24 or more hours. As can readily be seen in the data, effects like reduction of the activity below the control/vehicle levels can be detected and add information to the end point estimation via the WST-1 vitality test. The onset of effects reducing or elevating cellular vitality may thus also be derived from the data presented here and add the temporal aspect of this temporally dynamic process.

### 3.4. Further Aspects and Outlook

We have characterized a limited set of PDE inhibitors and autophagy modulators in SCNCRE cells in a specific setup quantifying transcriptional activity in a reporter gene assay and have gained some insight into the relevant effects of the PDEs expressed in these cells.

That 3-MA dose-dependently elevated CRE-luc activity with an EC_50_ at approximately 1–3 mM was a surprising finding. It was also surprising that at these concentrations, this cell line shows signs of diminishing vitality. Thus, it has to be taken into account that a frequently used and obviously highly useful research reagent like 3-MA may exert surprising effects in the future, especially in newly designed experimental setups. It may also explain some previously observed, but so far unexplained effects and should be taken into account for future ventures using this substance.

### 3.5. Limitations

Among the factors that are potentially important for some of the minor, not statistically significant findings are vehicle effects, especially those of DMSO. SCNCRE cells are particularly vulnerable to DMSO, especially if applied purely from stock solutions. Thus, the maximal final amount of DMSO was almost always below 1% *v*/*v*. The one condition where this could not be realized was the combination of dexamethasone and RU-486 (Figure 4), so this might be an explanation for the reduced vitality under this condition after 6–8 h. 3-MA stocks were dissolved in water or cell culture medium (L-15/DMEM). 3-MA should be soluble in water up to 200 mM, but we never reached a higher stock solution than 100 mM. This limited the dose–response curve to a final 3-MA level of 30 mM, which may influence cell reactions by osmolar effects on top of diverse pharmacological (PI3K /PDE-inhibition) ones. Finally, the plasma membrane passage time (the applied molecule may be amphiphilic and stay in the membrane for different amounts of time) and the target location inside the cell, where target proteins may attach to the inner (cytosolic) part of the plasma membrane, may diffuse in the cytoplasm or locate to other compartments of the cells like caveoli (membrane cavities) or organelles. All these latter processes may influence the types of measurements of the kind s we performed in the present work.

## 4. Materials and Methods

### 4.1. Cell Culture

Stable clones of CRE-luciferase (CRE-luc)-expressing immortalized mouse SCN neurons (named SCNCREs) frozen at a density of 1.5 million/mL/vial at −80 °C in freezing medium (IBIDI, Munich, Germany) thawed from the original mixed clones were used in up to 12 passages for the experiments. SCNCRE cells were passaged once a week at a density of 1 million cells per 75 cm of flask as described in previous studies [2,3].

### 4.2. Determination of Luminescence Activity

CRE-dependent luminescence activity was measured as described [2,3]. Briefly, 20,000 cells per well of a 96-well plate were plated out in a 100 µL volume and left in the incubator overnight to promote adhesion. Experiments were performed in 200 µL of medium/well containing 0.5 mM of Luciferin and measured in a luminometer (Berthold Centro LB960 purchased from Berthold Technologies GmbH & Co. KG, Bad Wildbad, Germany) at 35° Celsius for 0.1 sec per well. Luminescence data are displayed as relative luminescence units (RLU). Cells were measured after different times of incubation (e.g., every 15 min for 24 or more hours) with and without chemical agents potentially influencing CRE-luciferase (CRE-luc) signaling if not indicated otherwise.

### 4.3. Determination of Cell Vitality

The WST-1-based colorimetric assay is widely used for the nonradioactive quantification of cell proliferation, cell viability, and cytotoxicity (Roche, Mannheim, Germany). After the CRE-luciferase activity measurements, the medium was changed to OptiMEM including 10% *v*/*v* WST-1 reagent, incubated for one hour and absorbance was determined at 450 nm in a 96-well plate spectrophotometer.

### 4.4. Data and Statistical Analysis

Raw luminescence data (relative luminescence units = RLU) were recorded every 15 min in a luminometer (Berthold Centro LB960). The mean and standard deviation of four recorded RLU values were determined using GraphPad Prism 8.3. For comparison of the dose-dependency, the “area under the curve” was used to integrate both the amplitude and duration of a response into one parameter. Alternatively, the mean and standard deviation of the values at a time of maximal amplitude were compared between the different molecules and their combinations. Where data were compared in terms of statistical analysis, we first confirmed a normal distribution, tested for statistical difference in error size and then used one way ANOVA with Sidak’s multiple comparisons test.

### 4.5. Materials

3-methyladenin (3-MA), Rapamycin, Isoproterenol and Tunicamycin were dissolved in water or cell culture medium. LY294002, Dexamethasone, corticosterone, RU-486, Rolipram, Zaprinast, Milrinone and IBMX were dissolved as stock solutions in dimethylsulphoxide (DMSO; all substances were from Sigma-Aldridge, Merck KGaA, Darmstadt, Germany) The purity was > 99% by HPLC. The Luciferin [39] was from Promega (Promega, Heidelberg, Germany) and was dissolved directly in cell culture medium at the indicated concentrations. The purity in the recent lot, 0000228134, was > 98.5% by HPLC as supplied. Reagents or appropriate vehicles were applied to the media for the indicated periods.

## Data Availability

The data are available via the authors.

## Supplementary Materials

The following supporting information can be downloaded at: https://www.mdpi.com/article/10.3390/molecules30153229/s1.
