# Supplementary material for: PDE Inhibitors and Autophagy Regulators Modulate CRE-Dependent Luciferase Activity in Neuronal Cells from the Mouse Suprachiasmatic Nucleus
Source: Molecules. 2025 Aug 1;30(15):3229. doi: 10.3390/molecules30153229 (PMC12348409; doi:10.3390/molecules30153229)
Supplement: Supplementary file 1 [file molecules-30-03229-s001.zip › Figure S1.pdf]

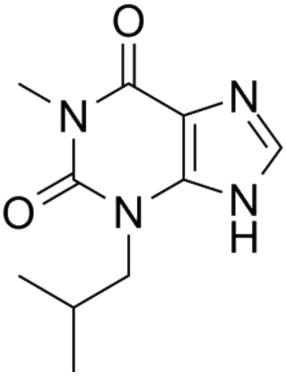

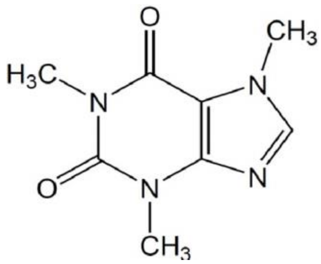

Caffeine

Isobuthyl-methyl-xanthine (IBMX)

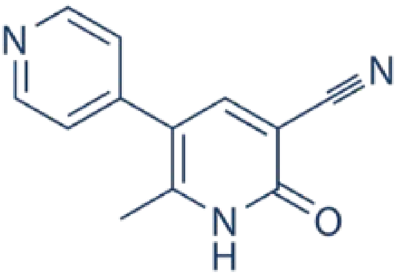

Milrinone (Win472043)

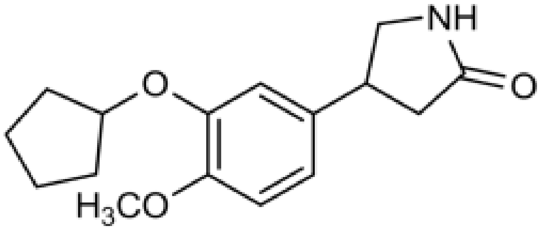

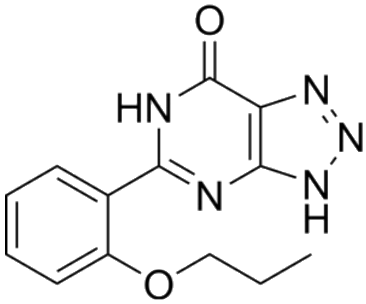

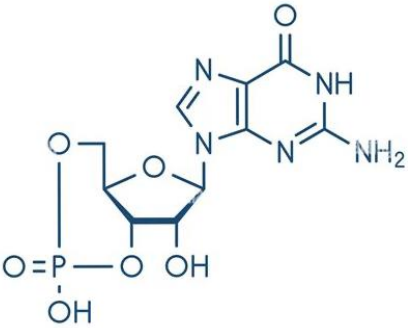

Rolipram

Zaprinast

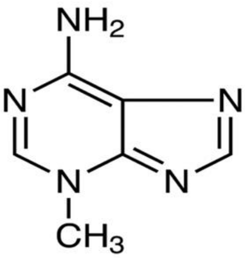

cGMP

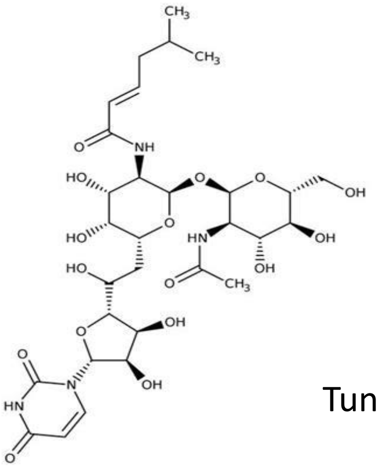

3-Methyladenin (3-MA)

Tunicamycin

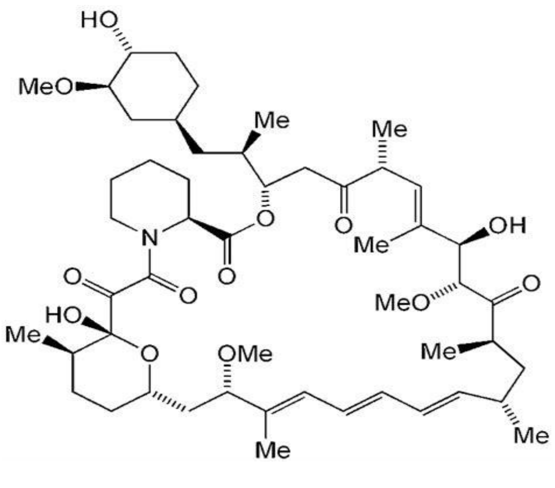

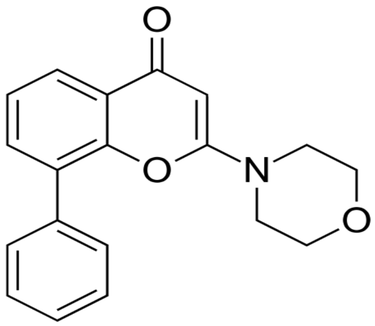

Rapamycin/Sirolimus

LY294002
