# Supplementary material for: PDE Inhibitors and Autophagy Regulators Modulate CRE-Dependent Luciferase Activity in Neuronal Cells from the Mouse Suprachiasmatic Nucleus
Source: Molecules. 2025 Aug 1;30(15):3229. doi: 10.3390/molecules30153229 (PMC12348409; doi:10.3390/molecules30153229)
Supplement: Supplementary file 1 [file molecules-30-03229-s001.zip › Figure S2.pdf]

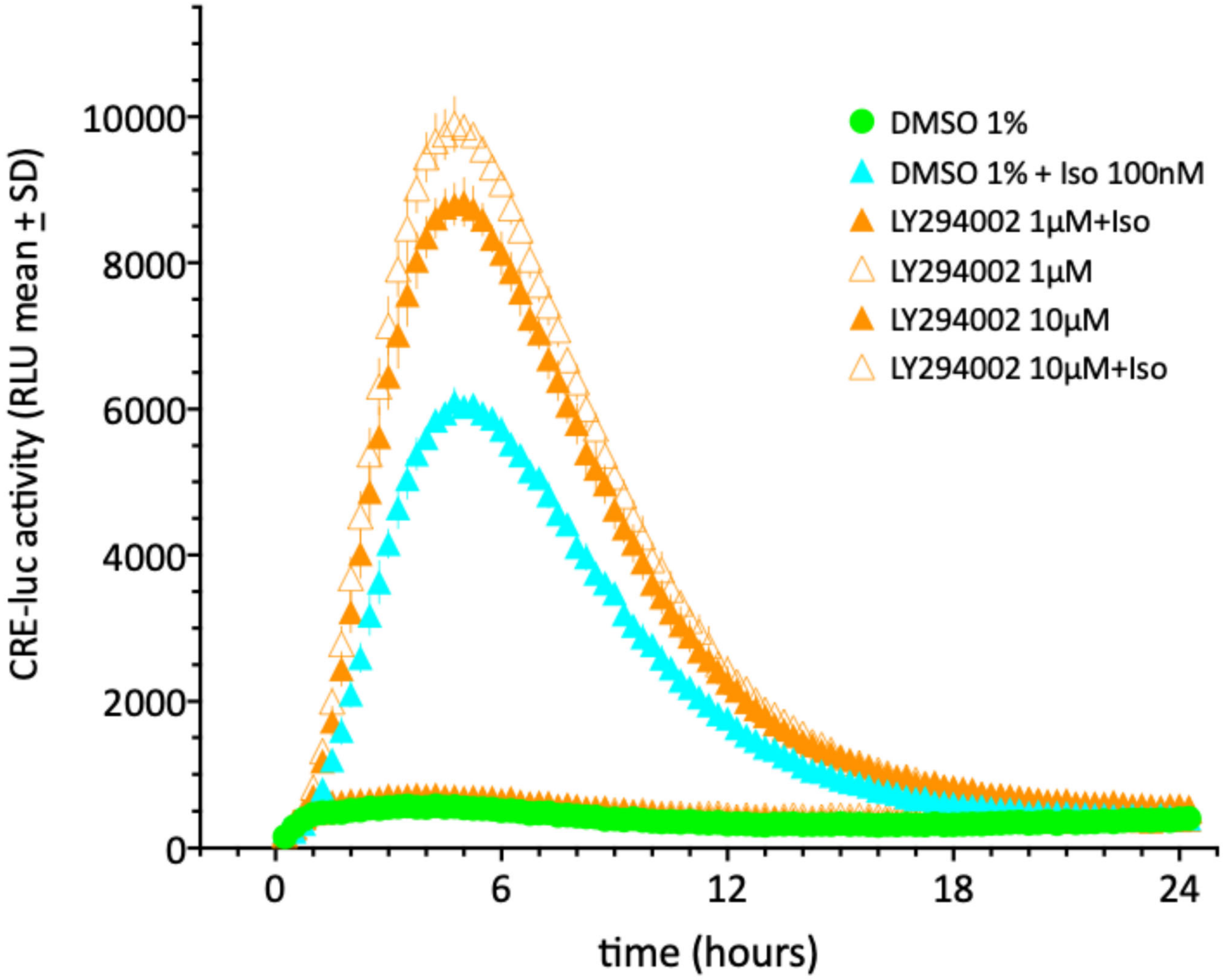

Supplementary Figure S2. CREluc activity after application of 1 or 10 µM of LY294002 alone or in combination with the beta-adrenoceptor agonist isoproterenol (100nM) in comparison to vehicle control (DMSO 1%). Shown are the means ± SD of N=4 equally treated single wells in 96 well multiwell plate. The insert shows the structure of LY294002.
