# Supplementary material for: PDE Inhibitors and Autophagy Regulators Modulate CRE-Dependent Luciferase Activity in Neuronal Cells from the Mouse Suprachiasmatic Nucleus
Source: Molecules. 2025 Aug 1;30(15):3229. doi: 10.3390/molecules30153229 (PMC12348409; doi:10.3390/molecules30153229)
Supplement: Supplementary file 1 [file molecules-30-03229-s001.zip › Figure S3.pdf]

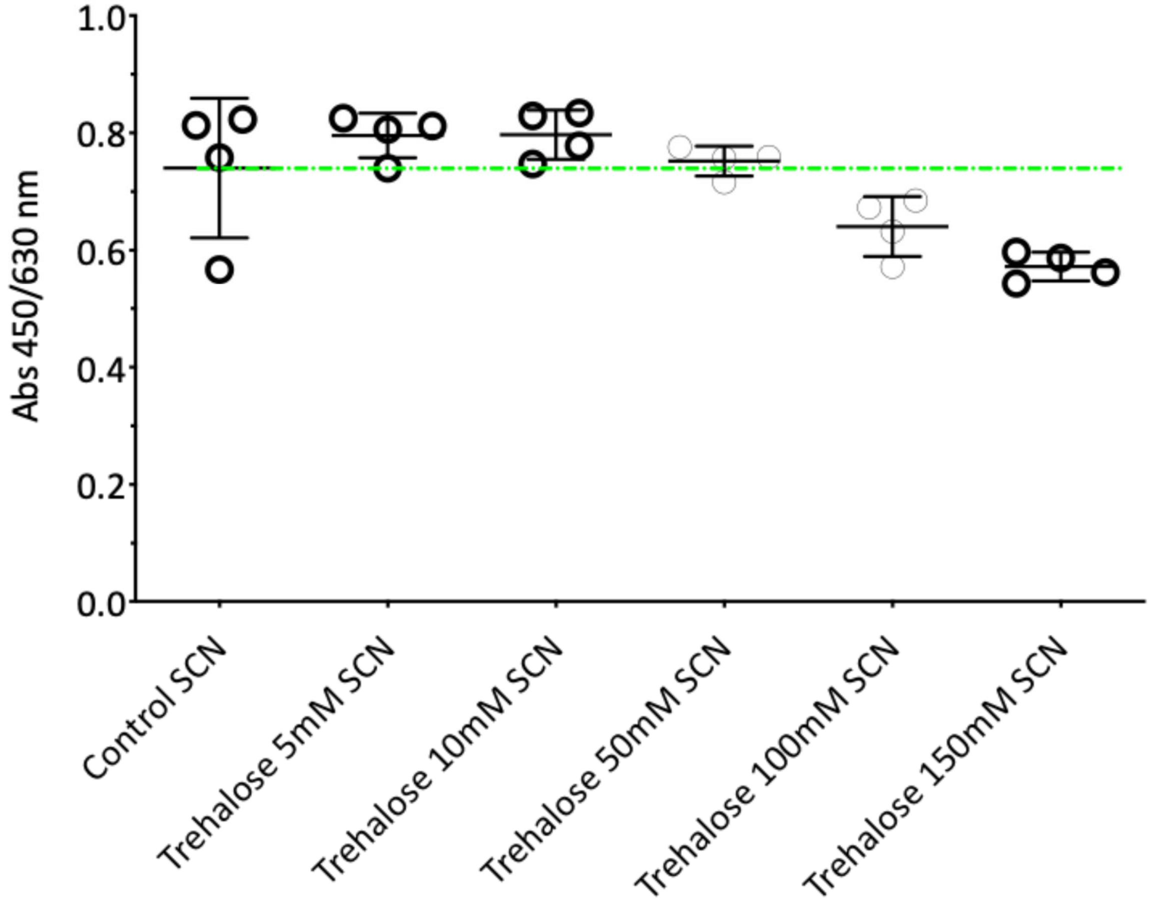

Supplementary Figure S3:
WST-1 in response to trehalose application. Shown are the means $\pm$ SD of N=4 equally treated single wells in 96 well multiwell plates. WST-1 levels are significantly reduced only at 100 and 150 mM as estimated by ANOVA with Dunnett`s post-test (see below). The green line represents the mean of control cultures.

Dunnett's multiple comparisons test	Mean Diff,	95,00% CI of diff,	Significant?	Summary	Adjusted P Value	A-?		
Control SCN vs. Trehalose 5mM SCN	-0,05550	-0,1716 to 0,06057	No	ns	0,5610	C	Trehalose 5mM SCN	
Control SCN vs. Trehalose 10mM SCN	-0,05675	-0,1728 to 0,05932	No	ns	0,5415	E	Trehalose 10mM SCN	
Control SCN vs. Trehalose 50mM SCN	-0,01175	-0,1278 to 0,1043	No	ns	0,9984	G	Trehalose 50mM SCN	
Control SCN vs. Trehalose 100mM SCN	0,09975	-0,01632 to 0,2158	No	ns	0,1064	I	Trehalose 100mM SCN	
Control SCN vs. Trehalose 150mM SCN	0,1680	0,05193 to 0,2841	Yes	**	0,0037	K	Trehalose 150mM SCN	
Test details	Mean 1	Mean 2	Mean Diff,	SE of diff,	n1	n2	q	DF
Control SCN vs. Trehalose 5mM SCN	0,7403	0,7958	-0,05550	0,04203	4	4	1,320	18
Control SCN vs. Trehalose 10mM SCN	0,7403	0,7970	-0,05675	0,04203	4	4	1,350	18
Control SCN vs. Trehalose 50mM SCN	0,7403	0,7520	-0,01175	0,04203	4	4	0,2796	18
Control SCN vs. Trehalose 100mM SCN	0,7403	0,6405	0,09975	0,04203	4	4	2,373	18
Control SCN vs. Trehalose 150mM SCN	0,7403	0,5723	0,1680	0,04203	4	4	3,997	18
