# Supplementary material for: PDE Inhibitors and Autophagy Regulators Modulate CRE-Dependent Luciferase Activity in Neuronal Cells from the Mouse Suprachiasmatic Nucleus
Source: Molecules. 2025 Aug 1;30(15):3229. doi: 10.3390/molecules30153229 (PMC12348409; doi:10.3390/molecules30153229)
Supplement: Supplementary file 1 [file molecules-30-03229-s001.zip › Figure S4.pdf]

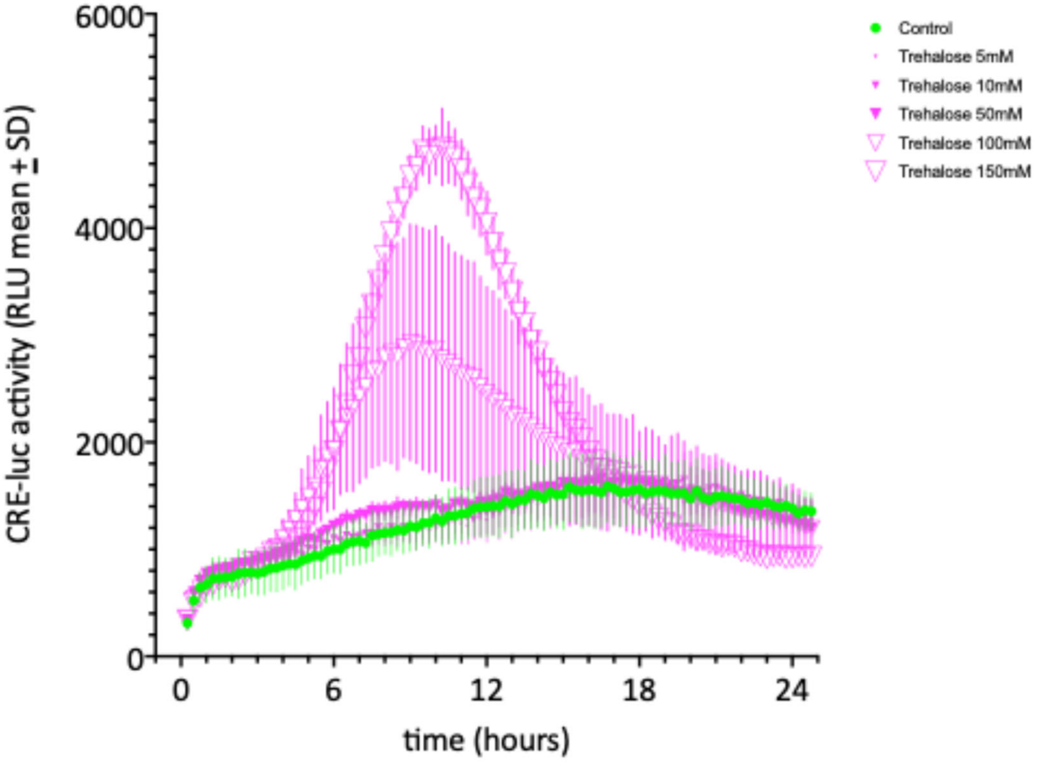


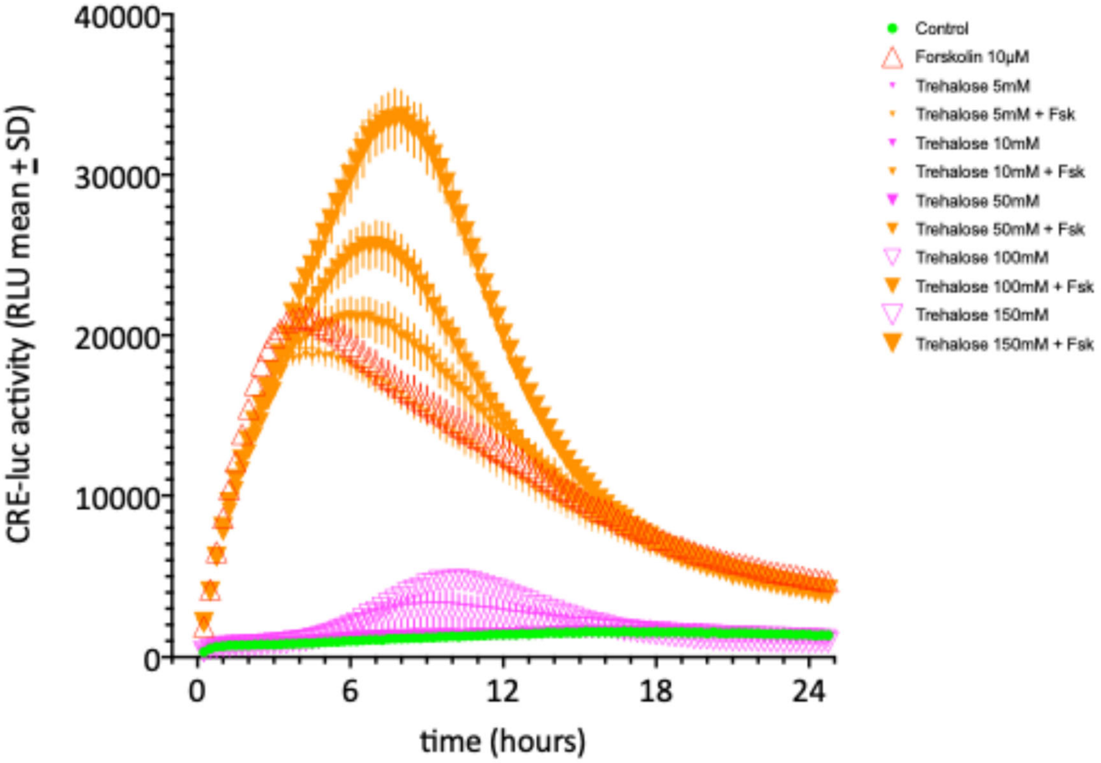


Supplementary Figure S4. SCNCREluc activity in response to trehalose alone (upper part) or trehalose in combination with forskolin (lower part). Shown are the means + SD of N=4 equally treated single wells in 96 well multiwell plates. WST-1 levels are significantly reduced only at 100 and 150 mM as estimated by ANOVA with Simaks post-test.
