# Supplementary material for: PDE Inhibitors and Autophagy Regulators Modulate CRE-Dependent Luciferase Activity in Neuronal Cells from the Mouse Suprachiasmatic Nucleus
Source: Molecules. 2025 Aug 1;30(15):3229. doi: 10.3390/molecules30153229 (PMC12348409; doi:10.3390/molecules30153229)
Supplement: Supplementary file 1 [file molecules-30-03229-s001.zip › File S1.pdf]

Number of families	1							
Number of comparisons per family	9							
Alpha	0,05							
Sidak's multiple comparisons test	Mean Diff,	95,00% CI of diff,	Significant?	Summary	Adjusted P Value			
3-MA 0.1mM vs. Control	-469,0	-2647 to 1709	No	ns	0,9989	K-M		
3-MA 0.3mM vs. Control	302,0	-1584 to 2188	No	ns	>0,9999	I-M		
3-MA 1mM vs. Control	9991	8105 to 11877	Yes	****	<0,0001	G-M		
3-MA 3mM vs. Control	71971	70085 to 73857	Yes	****	<0,0001	E-M		
3-MA 10mM vs. Control	220339	218453 to 222225	Yes	****	<0,0001	C-M		
3-MA 30mM vs. Control	9941	8055 to 11827	Yes	****	<0,0001	A-M		
3-MA 30mM vs. 3-MA 10mM	-210398	-211938 to -208858	Yes	****	<0,0001	A-C		
3-MA 30mM vs. 3-MA 3mM	-62030	-63570 to -60490	Yes	****	<0,0001	A-E		
3-MA 30mM vs. 3-MA 1mM	-50,00	-1590 to 1490	No	ns	>0,9999	A-G		
Test details	Mean 1	Mean 2	Mean Diff,	SE of diff,	n1	n2	t	DF
3-MA 0.1mM vs. Control	6077	6546	-469,0	746,3	4	4	0,6285	41
3-MA 0.3mM vs. Control	6848	6546	302,0	646,3	8	4	0,4673	41
3-MA 1mM vs. Control	16537	6546	9991	646,3	8	4	15,46	41
3-MA 3mM vs. Control	78517	6546	71971	646,3	8	4	111,4	41
3-MA 10mM vs. Control	226885	6546	220339	646,3	8	4	340,9	41
3-MA 30mM vs. Control	16487	6546	9941	646,3	8	4	15,38	41
3-MA 30mM vs. 3-MA 10mM	16487	226885	-210398	527,7	8	8	398,7	41
3-MA 30mM vs. 3-MA 3mM	16487	78517	-62030	527,7	8	8	117,6	41
3-MA 30mM vs. 3-MA 1mM	16487	16537	-50,00	527,7	8	8	0,09475	41

Statistics for Figure 2A CRE-luc

Number of families	1							
Number of comparisons per family	7							
Alpha	0,05							
Sidak's multiple comparisons test	Mean Diff,	95,00% CI of diff,	Significant?	Summary	Adjusted P Value			
3-MA 30mM vs. Control	-0,1559	-0,1780 to -0,1337	Yes	****	<0,0001	A-M		
3-MA 10mM vs. Control	-0,1313	-0,1534 to -0,1091	Yes	****	<0,0001	C-M		
3-MA 3mM vs. Control	-0,06675	-0,08891 to -0,04459	Yes	****	<0,0001	E-M		
3-MA 1mM vs. Control	-0,03825	-0,06041 to -0,01609	Yes	***	0,0001	G-M		
3-MA 0.3mM vs. Control	-0,01238	-0,03453 to 0,009783	No	ns	0,5994	I-M		
3-MA 0.1mM vs. Control	0,02025	-0,005336 to 0,04584	No	ns	0,1976	K-M		
3-MA 30mM vs. 3-MA 10mM	-0,02463	-0,04272 to -0,006533	Yes	**	0,0029	A-C		
Test details	Mean 1	Mean 2	Mean Diff,	SE of diff,	n1	n2	t	DF
3-MA 30mM vs. Control	0,1751	0,3310	-0,1559	0,007848	8	4	19,86	41
3-MA 10mM vs. Control	0,1998	0,3310	-0,1313	0,007848	8	4	16,72	41
3-MA 3mM vs. Control	0,2643	0,3310	-0,06675	0,007848	8	4	8,505	41
3-MA 1mM vs. Control	0,2928	0,3310	-0,03825	0,007848	8	4	4,874	41
3-MA 0.3mM vs. Control	0,3186	0,3310	-0,01238	0,007848	8	4	1,577	41
3-MA 0.1mM vs. Control	0,3513	0,3310	0,02025	0,009062	4	4	2,235	41
3-MA 30mM vs. 3-MA 10mM	0,1751	0,1998	-0,02463	0,006408	8	8	3,843	41

Statistics for Figure 2B WST-1

Sidak's multiple comparisons test	Mean Diff,	95,00% CI of diff,	Significant?	Summary	Adjusted P Value			
Control vs. Isoproterenol 100nM	-15053	-18084 to -12022	Yes	****	<0,0001	A-B		
Control vs. Rapamycin 10µg/ml	-53,00	-3084 to 2978	No	ns	>0,9999	A-K		
Isoproterenol 100nM vs. Rapamycin 10µg/ml	15000	11969 to 18031	Yes	****	<0,0001	B-K		
Isoproterenol 100nM vs. Rapa+Iso	1268	-1763 to 4299	No	ns	0,6746	B-L		
Test details	Mean 1	Mean 2	Mean Diff,	SE of diff,	n1	n2	t	DF
Control vs. Isoproterenol 100nM	6986	22039	-15053	1037	4	4	14,52	12
Control vs. Rapamycin 10µg/ml	6986	7039	-53,00	1037	4	4	0,05112	12
Isoproterenol 100nM vs. Rapamycin 10µg/ml	22039	7039	15000	1037	4	4	14,47	12
Isoproterenol 100nM vs. Rapa+Iso	22039	20771	1268	1037	4	4	1,223	12

Statistics for Figure 3A

Sidak's multiple comparisons test	Mean Diff,	95,00% CI of diff,	Significant?	Summary	Adjusted P Value			
Control vs. Isoproterenol 100nM	-15053	-17957 to -12149	Yes	****	<0,0001	A-B		
Control vs. Tunicamycin 2,5µM/ml	353,0	-2551 to 3257	No	ns	0,9982	A-M		
Control vs. Tun+Iso	-15047	-17951 to -12143	Yes	****	<0,0001	A-N		
Isoproterenol 100nM vs. Tunicamycin 2,5µM/ml	15406	12502 to 18310	Yes	****	<0,0001	B-M		
Isoproterenol 100nM vs. Tun+Iso	6,000	-2898 to 2910	No	ns	>0,9999	B-N		
Test details	Mean 1	Mean 2	Mean Diff,	SE of diff,	n1	n2	t	DF
Control vs. Isoproterenol 100nM	6986	22039	-15053	954,0	4	4	15,78	12
Control vs. Tunicamycin 2,5µM/ml	6986	6633	353,0	954,0	4	4	0,3700	12
Control vs. Tun+Iso	6986	22033	-15047	954,0	4	4	15,77	12
Isoproterenol 100nM vs. Tunicamycin 2,5µM/ml	22039	6633	15406	954,0	4	4	16,15	12
Isoproterenol 100nM vs. Tun+Iso	22039	22033	6,000	954,0	4	4	0,006289	12

Statistics for Figure 3B

Sidak's multiple comparisons test	Mean Diff,	95,00% CI of diff,	Significant?	Summary	Adjusted P Value			
Control vs. Isoproterenol 100nM	-15053	-17416 to -12690	Yes	****	<0,0001	A-B		
Control vs. Dexametason 100nM	2480	117,0 to 4843	Yes	*	0,0356	A-C		
Control vs. RU486 100 nM	1197	-1166 to 3560	No	ns	0,6810	A-E		
Control vs. RU486+Dex	3100	737,0 to 5463	Yes	**	0,0054	A-G		
Isoproterenol 100nM vs. Dex+Iso	11049	8686 to 13412	Yes	****	<0,0001	B-D		
Isoproterenol 100nM vs. RU486+Iso	-2176	-4539 to 187,0	No	ns	0,0844	B-F		
Isoproterenol 100nM vs. RU486+Dex+Iso	1643	-720,0 to 4006	No	ns	0,3154	B-H		
Test details	Mean 1	Mean 2	Mean Diff,	SE of diff,	n1	n2	t	DF
Control vs. Isoproterenol 100nM	6986	22039	-15053	806,1	4	4	18,67	24
Control vs. Dexametason 100nM	6986	4506	2480	806,1	4	4	3,076	24
Control vs. RU486 100 nM	6986	5789	1197	806,1	4	4	1,485	24
Control vs. RU486+Dex	6986	3886	3100	806,1	4	4	3,846	24
Isoproterenol 100nM vs. Dex+Iso	22039	10990	11049	806,1	4	4	13,71	24
Isoproterenol 100nM vs. RU486+Iso	22039	24215	-2176	806,1	4	4	2,699	24
Isoproterenol 100nM vs. RU486+Dex+Iso	22039	20396	1643	806,1	4	4	2,038	24

Statistics for Figure 4
